# ‘First of all, I need training’: a qualitative study evaluating the Fiji community health worker training program

**DOI:** 10.1186/s12875-024-02480-8

**Published:** 2024-06-26

**Authors:** Samuel Thio, Azeb Gebresilassie Tesema, Bindu Patel, Unise Vakaloloma, Colleen Wilson, Rohina Joshi

**Affiliations:** 1 School of Population Health, University of New South Wales, Sydney, Australia; 2 The George Institute for Global Health, Sydney, Australia; 3 Centre for the Prevention of Obesity and Non-Communicable Diseases, Fiji National University, Suva, Fiji; 4 Ministry of Health and Medical Services, Suva, Fiji; 5 The George Institute for Global Health, New Delhi, India

**Keywords:** Community health worker, Qualitative study, Training program, Republic of Fiji

## Abstract

**Background:**

Fiji faces a growing burden of diseases and a significant emigration of health workers, heightening the role of community health workers (CHWs) in healthcare delivery. Effective training is crucial for CHWs to enhance their capacity and service quality. This study evaluates CHW training in Fiji, aiming to identify areas for improvement.

**Methods:**

A qualitative study was conducted, encompassing a review of national policies on CHW training, six focus group discussions, and interviews with CHWs and their supervisors across Fijian subdivisions. This study was collaboratively designed with Fiji's Ministry of Health and Medical Services (MOHMS). Data was transcribed, coded, and thematically analysed using the Community Health Workers Assessment and Improvement Matrix (CHW-AIM).

**Findings:**

While CHW training policies in Fiji are well-established, discrepancies exist between the policy and its implementation. Challenges include inconsistent training for new recruits, limited resources, and variability in training content and frequency of training across divisions, especially concerning noncommunicable disease (NCD) training.

**Interpretation:**

To enhance the CHW training program in Fiji, a restructuring and standardisation of both pre-service and in-service training is necessary, tailored to the needs of each division. Investment in ongoing capacity building, alongside the development and revision of training guidelines, particularly for managing NCD complications in the community, is crucial. Implementing these changes will enable CHWs in Fiji to be better equipped for providing essential community-based care.

**Supplementary Information:**

The online version contains supplementary material available at 10.1186/s12875-024-02480-8.

## Introduction

Republic of Fiji (Fiji) confronts a triple burden of infectious diseases, non-communicable diseases (NCDs), and injuries, compounded by the looming threat of climate change [1, 2]. Predominant causes of death include diabetes (19.7%), ischemic heart diseases (16.6%), and cerebrovascular diseases (7.4%) [3]. The World Health Organization (WHO) recommends a minimum of 4.45 health workers per 1000 population to achieve universal healthcare coverage [4]. Fiji, however, faces a significant healthcare workforce challenge, with a low density of 0.9 practising physicians per 1000 population as of 2016 [2, 5, 6]. This issue is exacerbated by the internal migration of healthcare workers from rural to urban areas and high emigration rates of doctors and nurses, leading to a strained public health system [7, 8].

Initiated in the 1980s, the Community Health Worker (CHW) program was a strategic response to this scarcity, especially in rural and maritime regions where healthcare services are most needed [9]. CHWs are involved in coordinating community health activities, reporting to the Ministry of Health and Medical Services (MOHMS) through nursing supervisors, and serving on village development committees [10]. They serve a vital role in linking the health system with the community helping zonal nurses on the local level [10].

CHWs, typically community-selected and predominantly women, undergo a 6-week specialised training, including mandatory professional development and in-service training [3, 5]. Their role encompasses first aid and basic treatment, supported by community-built dispensaries and the MOHMS-provided training, medicine, and equipment [5].

Despite initial successes, the program's momentum has waned over the years, with the number of active CHWs dropping from around 3,000 in the 1990s to 1,805 in 2015 [3]. Moreover, a 2011 situational analysis by the Fiji Health Sector Support Programme revealed a lack of standardised training for CHWs, with inconsistent duration and training topics [10]. This concerning trend align with a recent governmental report, revealing that 44% of the 1643 active CHWs lack formal training [11]. Yet, no comprehensive study has elucidated the barriers to CHW training in Fiji.

This study aims to bridge this gap by examining the translation of CHW training policy into practice. Through qualitative methods, we explore the implementation of CHW training, identify policy-practice discrepancies and enablers and barriers to effective CHW training in Fiji (Table 1).

**Table 1:** Context

Fiji, a highly urbanised state among the Pacific Island countries, has over half (51%) of its population living in urban areas [12]. The country consists of 332 islands, is home to 893,468 people, and boasts a multicultural and multi-religious demographic. Ethnic Fijians (iTaukei) make up 57% of the population, followed by 37% Indo-Fijians, with the remainder comprising Chinese, Caucasians, and other ethnicities [13–15].
Public health services in Fiji are divided into four divisions - Northern, Western, Eastern, and Central. Primary healthcare and tertiary services rely on public funding, but challenges such as low tax levels, austerity measures, and funding cuts have impeded health improvements [5]. The program underwent a revamp funded by Australian Aid, aiming to strengthen primary care delivery by Nursing Stations and Health Centres, positioning trained CHWs as pivotal links between the community and healthcare system [2, 10].
CHW Selection: As outlined in the 2015 Community Health Worker Policy [10], the CHWs are to be selected by their own communities with consultation from zone nurses and endorsed by the MOHMS and satisfy various criteria, including:
- Demonstrates long-term commitment to stay in the community and has an interest in health and development issues. Willing to make household visits.
- Mature and respected member of the community.
- Honest, friendly, and possesses good communication skills and ability to maintain confidentiality.
- Attained a minimum education level (Form 4 for urban/peri-urban areas or Form 2 for rural/remote areas).
- Has supportive consent of partner and immediate family.
- Knowledgeable in spoken and written English for CHWs serving informal communities.

## Methods

### Study design

We conducted a qualitative study combining policy review, semi-structured interviews, and focus group discussions (FGDs) to capture the CHWs’ perspectives on training programs and supervision practices, and their roles within the healthcare system from January to March 2023.

### National policy review

A comprehensive desktop review of Fiji's national policies and documents related to CHWs was conducted using the modified Community Health Worker Assessment and Improvement Matrix (CHW-AIM) (Appendix 3). A comprehensive review of publications and reports helped us identify relevant policies, this included the National Community Health Worker Policy (2015), MOHMS annual reports, and official CHW training manuals. Additionally, studies evaluating CHW training and experiences were reviewed to understand the established training policies and programs for CHWs in Fiji.

### Qualitative study

#### Participant recruitment and data collection

Data was collected across six sub-divisions representing Fiji's four administrative divisions. We conducted six FGDs with 41 CHWs and conducted six interviews with nurse supervisors. The participants were purposively recruited from selected sub-divisions with the assistance of the Chief Nursing and Midwifery Officer. CHWs were recruited for the FGDs if they were actively providing primary health care services in their community. Nurse supervisors were selected if they were actively involved in supervising one or more CHWs and in managing both nurses and CHWs in the community. All the participants agreed to be interviewed, with no dropouts.

The CHW-AIM toolkit guided the development of the FGD and interview guides (Supplementary file 1), which were used to interview CHWs and supervisors. For CHW, questions covered themes such as the recruitment and role of CHWs, training, equipment and supplies, and supervision. For supervisors, questions explored the role of CHWs, training, equipment and supplies, supervision, individual performance evaluation, incentives, and opportunities for advancement. The FGDs and interviews were conducted in English and audio taped by two of the researcher members (RJ and BP) and a researcher from Fiji (UV). Interviews were conducted in quiet places within the health facilities. The interviews lasted approximately 40 minutes, while the FGDs lasted 80-90 minutes on average. This process also ensured data saturation.

#### Data analysis

For the policy review, key documents were synthesised narratively to summarise the content related to the CHW program and training. For the qualitative analysis, interviews were transcribed verbatim, supplemented by interviewer field notes. The transcriptions were initially coded by researchers ST and AGT, with subsequent manual coding in Microsoft Excel by ST. Key themes and sub-themes were extracted and discussed within the research team. Policymaker feedback was sought to contextualise and validate the findings. Data analysis involved a triangulation approach, integrating findings from the policy review, FGDs, and interviews. Themes identified through the CHW-AIM framework analysis of the policy documents were further explored and validated through the qualitative data from the FGDs and interviews. Data source triangulation involved comparing data within and across different sources to identify key themes. According to Carter et al. (2014), using mixed data source triangulation can lead to a broader understanding of the topic of interest. We reported the results in accordance with the consolidated criteria for reporting qualitative research (COREQ) (Supplementary file 2).

## Results

### Section 1: policy document review outcomes

In 2013, Fiji established a formalised CHW program to unify CHW training, encompassing core competencies and modules in first aid, child health, safe motherhood, and wellness [10]. The Fiji Health Sector Support Programme (FHSSP) crafted four training manuals. The 2015 Community Health Worker Policy by the MOHMS outlined the development of Standard Operating Procedures for CHWs, highlighting governance, training, selection, financing, incentivisation, and integration into the health system. A pivotal goal was fostering collaboration with stakeholders and communities to enhance CHW program development and promote CHW-community health ownership [10].

The policy advocated establishing a National Health Workers Steering Committee (NCHWSC) for CHW governance. It specified MOHMS as the primary funding source, guided by NCHWSC recommendations and was subject to annual review [10].

Key policy directives included: 1) Enhancing community ownership and advocating a holistic, stakeholder-inclusive approach in the CHW program; 2) Upholding approved standards in primary healthcare service delivery; 3) Embracing national values of universal access, equity, quality, and social justice. The 2020-25 MOHMS strategic plan integrates CHWs in its vision to fortify and decentralise clinical services, aiming to curb communicable and non-communicable diseases through community-based care [5].

The CHW training spans six weeks, blending theoretical and practical components, including hospital attachments, culminating in an examination and certification. As of the review, eight training modules were available: Core Competencies (2013), Tuberculosis Control (2013), Safe Motherhood (2014), Wellness Promotion (2014), Child Health (2014), Emergency Training (2015), Rheumatic Heart Disease (2018), and Palliative Care (2022) (Appendix 1).

Community health workers are nominated by their village committees and undergo a formal selection process involving the submission of documentation to supervising zone nurses. National policy mandates that the sub-divisional nurse manager (SDNM) is responsible for the training of CHWs. The training of CHWs is meant to be standardized across Fiji, with the MOHMS providing training materials and curriculum. The policy also outlines a supervisory structure for CHWs, emphasizing regular reporting and communication with their supervising zone nurses which involves monthly reports, community visits and phone consultations.

### Section 2: results from the Fgds FGDs and interviews

Our qualitative study involved forty-one CHWs participating in FGDs across four divisions: eight from the Eastern Division (Levuka), nine from the Northern Division (Seaqaqa), seven from the Central Division (Nausori and Valelevu), and seventeen from the Western Division (Balevetu and Sigatoka). Additionally, we conducted in-depth interviews with six female supervisors, all experienced subdivisional nurse managers (SDNMs) overseeing the subdivision, including zonal nurses and CHWs.

The CHWs predominantly identified as female (93%), with a minority of 7% identifying as male. Their ages ranged from 30 to 60 years, and all resided within the communities they served. The majority (54%) had been working as CHWs for over six years, 24% for 2-6 years, and 22% for less than two years. Regarding educational background, 68% completed secondary education, 28% had college education, 5% had primary education, and 2% held postgraduate qualifications. Detailed participant characteristics are presented in Appendix 2.

Training experiences varied among the divisions. In the Eastern Division, three out of eight CHWs received training in 2013. Most CHWs in the Northern and Western Divisions attended training sessions in 2022, while all CHWs in the Central Division participated in recent training in 2023. These details are summarised in Table 2.

**Table 2:** Characteristics of participants in CHW FGDs (*n*=41)

Socio-demographic Variables	Frequency (%)
**Years Worked as CHW**	
Less than 2 years	9 (22%)
2-6 years	10 (24%)
More than 6 years	22 (54%)
**Regional Division**	
Eastern	8 (20%)
Northern	9 (22%)
Western	17 (41%)
Central	7 (17%)
**Last Training Session Attended**	
No Training	6 (15%)
2013	3 (7%)
2022	22 (54%)
2023	7 (17%)
No Answer	3 (7%)
**Highest Education Level**	
Primary	2 (5%)
Secondary	28 (68%)
Tertiary	10 (24%)
Postgraduate	1 (2%)

As shown in Table 3, our analysis identified four themes and sub-themes revealing critical insights into the training and experiences of CHWs in Fiji. Some quotes from each theme are also presented in Appendix 4.

**Table 3:** Themes and sub-themes of the study

Themes	Sub-themes
Theme 1: Variability in the Initial Training of CHWs	Inconsistency of initial training
Theme 2: Variability in Training Content, Duration, and Providers for CHWs	Regional Variations in Training Quality and Content
	Inconsistency in Training Duration
	Diverse Training Providers
Theme 3: Supervision as a Means for Capacity Building and Evaluation	Monthly Reports and Evaluations
	Attachments at Hospitals and Clinics
	Refresher Training
	Ongoing Supervision
Theme 4: Recommendations from Participants	Standardised Training for CHWs
	Priority Topics and Training Materials
	Career Progression and Recognition
	Transportation Support for Training
	Development of a CHW Evaluation Checklist
	Review of the CHW Training Program

### Theme 1: variability in the initial training of CHWs

The adherence to the formal six-week training program, as documented in the 2015 Fiji CHW policy, was found to be inconsistent. CHWs recruited over a decade ago generally received this comprehensive training. A participant from a Western subdivision reflected on their experience, stating, *“We were lucky that time... we went through family planning, everything government and for six weeks...”* (FGD1 Balevetu). This training encompassed various modules, including first aid, dressings, core competency, diabetes management, safe motherhood, and child health, culminating in certification.

However, newer CHWs, particularly those who joined during the pandemic, often lacked this extensive training. This discrepancy was notably pronounced in the Eastern division, where a participant lamented, *“Here we are just named as community health workers, but we don’t get training”* (FGD7 Levuka). Training duration for most participants was less than six weeks, contradicting policy guidelines. For instance, Eastern division participants received only one week of training in 2013, a pattern echoed in the Central division in 2017. In contrast, a participant from the Western division recalled receiving a month-long training in Suva.

The responsibility of identifying training needs falls on zone nurses, while sub-divisional nurses are tasked with leading the training sessions. Nevertheless, training has been sporadic in recent years, as highlighted by a nurse manager: *“...since my three years here, I haven't conducted any training”* (SDNM4). The impact of the COVID-19 pandemic was also mentioned as a factor in the training deficit: *“Maybe because of COVID, some of them [CHWs] were not yet trained”* (SDNM7).

### Theme 2: variability in training content, duration, and providers for CHWs

The 2015 Fiji CHW policy, though providing a framework for CHW training, is implemented with variations in content, duration, and the training provider, especially across different regions.

#### Regional variations in training quality and content

Training consistency varied significantly among CHWs. Those in the Central and Western divisions generally received more extensive training compared to their counterparts in the Eastern and Northern divisions. A notable range of training modules were covered, including core competencies, safe motherhood, wellness promotion, first aid, CPR, and more specialised areas like palliative care, emergency management, and disease-specific modules.

A Central subdivision supervisor highlighted the depth of training in their region, *"In the training, they [CHWs] were taught how to take blood pressure and understand normal and abnormal readings"* (SDNM4). Conversely, CHWs in the Eastern subdivision mainly received training on standard modules without additional workshops.

#### Inconsistency in training duration

The number of training days varied, with some supervisors reporting condensed training sessions. For example, a Western subdivision supervisor mentioned organising a three-day annual training, with the last day focused on practical skills (SDNM1).

#### Diverse training providers

The MOHMS, Fiji Cancer Society, and the Red Cross emerged as key training providers. Each brought unique strengths to the training process. For instance, the Red Cross was consistently involved in training across all four subdivisions, especially in CPR, first aid, and workshops on noncommunicable diseases (NCDs) and tuberculosis (TB).

The Fiji Cancer Society played a crucial role in providing palliative care training, particularly in the Central and Western subdivisions. This training equipped CHWs with essential skills for caring for bedridden patients, including personal hygiene and comfort measures. One participant from Valelevu shared,

*"We learned to care for bedridden patients, including changing diapers and bathing them, through the Cancer Society's workshop"* (FGD5 Valelevu).

### Theme 3: supervision as a means for capacity building and evaluation

#### Monthly reports and evaluations

CHWs are required to complete monthly reports detailing community profiles, which are then submitted to zone nurses. These reports are crucial for evaluation and feedback, serving as a basis for performance assessment. A subdivisional nurse manager explained, *“All community health workers come under one zone nurse... They work with the zone nurse... then I will check, verify, and send to headquarters”* (SDNM1). The incentive structure for CHWs is also linked to these reports, as highlighted by a participant: *“No report, no allowance”* (FGD3 Seaqaqa).

#### Attachments at hospitals and clinics

Particularly in the Central subdivision, CHWs have opportunities for on-the-job training through regular attachments at hospitals, outpatient departments, and nursing stations. This hands-on experience is seen as invaluable for their skill development. A participant described their routine: “*Every Wednesday, we usually come to the clinic and do attachment there... and that’s where we learn all this”* (FGD4 Nausori). Supervisors also noted the benefits of these attachments in providing practical learning experiences for CHWs.

#### Refresher training

Monthly meetings between nurses and CHWs serve as platforms for refresher training on various topics tailored to the needs identified in CHW reports. These sessions cover a range of subjects, from reporting and community profiling to communicable and noncommunicable disease management. A nurse supervisor mentioned, *“We hold meetings end of the month when they bring their reports, and that's how we brief them”* (SDNM4).

#### Ongoing supervision

CHWs often seek advice from zone nurses or supervisors via phone, using apps like Viber or Facebook Messenger for communication. This remote support is supplemented by periodic community visits by zonal nurses, although the frequency of these visits varies across regions. This could vary from 1-2 times a month to once every 3 months.

### Theme 4: recommendations from participants

#### Standardised training for CHWs

Participants voiced a strong need for more comprehensive training, particularly for new CHWs who began their roles during the pandemic. One CHW from the Eastern Division emphasised the urgency of this need: *“...I need training… As soon as possible…”* (FGD4 Eastern Division). Refresher workshops to update and enhance their skills were also suggested. A participant from Nausori expressed, *“I just need more workshops like that so we can be more refreshed”* (FGD4 Nausori).

#### Priority topics and training materials

Learning about NCDs emerged as a recurring priority, reflecting the high prevalence of NCD cases in communities as one CHW described: *“… Because there’s plenty of sickness on NCDs.”* (FGD7 Levuka). CHWs also expressed a desire for accessible training manuals for reference in their practice, *“I would like to get some more [training] manuals...”* (FGD7 Levuka).

#### Career progression and recognition

Participants expressed aspirations for career advancement and recognition, suggesting the provision of identity cards and certificates. This was seen as a way to validate their training and potentially pave the way for roles as mid-level health providers, attracting younger community members to the CHW role. As a Nausori participant put it: *“All we need is the training and at least the certificate to be there as proof. Because in the [community], like my experience, they always need proof.”* (FGD4 Nausori)*.*

#### Transportation support for training

The lack of transportation funding was highlighted as a barrier to attending training sessions. Participants often had to self-fund their travel to workshops, as noted by a participant from Valelevu: *“...every time we’ll like, they’ll write no bus fare, you have to find your own transport…”* (FGD5 Valelevu).

#### Development of a CHW evaluation checklist

Supervisors suggested a standardised competency checklist to facilitate more objective evaluations and improve the reporting and management of CHWs’ training.

As described by a supervisor:

*“Yes, I do feel there is still a lot of room for us to improve, we have to work on the reporting system, from our level to the National level. They [MOHMS] need to work on standardising their [CHWs] core-competencies… or at least to evaluate what they’ve learned and compare it with the work they’ve been doing…”* (SDNM6).

#### Review of the CHW training program

Our findings indicate a crucial need for revising the CHW training program in Fiji. The aim should be to standardise the training modules and integrate core competencies effectively. A key suggestion from the stakeholders is the consolidation of the current array of training manuals into a single, comprehensive guide. This would make the training material more accessible and manageable for CHWs. As one subdivisional nurse manager expressed, the goal is to create a resource that is both comprehensive and understandable, tailored to the educational level of the CHWs: *“There's an effort to compress the material into one detailed yet straightforward manual for better comprehension by the CHWs”* (SDNM4).

## Discussion

The national policy documents relating to the CHW program suggest that the CHW training program is functional (in accordance with the CHW assessment and improvement matrix, 2018) in the country [10, 16]. However, based on the interviews and group discussions, there is a policy-implementation gap wherein the CHW training varies between partially functional and non-functional mainly due to the variation in training amongst CHWs (Appendix 3) [16].

We found that the newer CHWs were unlikely to have undergone formal training this is supported by the our document review which revealed a concerning statistic from a recent government report: 44% of the 1,643 active CHWs in Fiji lack formal training [11]. One reason for the lack of training for the newly recruited CHWs may be the COVID pandemic, as the pandemic disrupts the education of health workers and the capacity of governments to provide training [17]. There were marked differences between the training and resources availability for CHWs in different regions. CHWs from the Central subdivision had been trained in all the modules, including practical sessions. They received additional training from the Fiji Cancer Society in the palliative care module and were trained to use blood pressure machines, dressings, and glucose monitoring strips. In contrast, most CHW participants from the Eastern subdivision had not received formal training besides first aid training with the Red Cross. The lack of standardised training also highlights the health inequity between Fiji's urban and rural remote centres, wherein communities residing in the central division had better access to care compared to those living in geographically isolated regions (where CHWs had not received training) [8].

Refresher workshops, hands-on training, upskilling, and additional supervision are important to CHW programs, as they improve CHW knowledge. In the context of CHW training in rural India, theory and practical training drastically improved CHW knowledge in NCDs [18]. Additionally, a systematic review by Abdel-All et al. (2018) demonstrated the need for ongoing education and training of CHWs for effective prevention and management of NCDs [19]. We found that CHWs also expressed the need for certifications and identity badges to substantiate their role in the community and give them evidence of qualifications. There was also a desire for a career progression pathway to upskill and work as a mid-level provider in health facilities. The CHW program in Ethiopia allows CHWs to gain training and progress to higher levels within the health system [20]. Such initiatives increase motivation, help with retention, and attract the next generation to work as CHWs [21].

Due to the rising prevalence of NCDs and the lack of community-based programs to address NCDs, most CHWs requested additional training to manage NCD cases, especially complications arising due to diabetes. This also demonstrated their ability to be responsive to community needs. Currently, there is no official NCD training module for CHWs. This is especially pertinent with the rise in NCDs, the country's triple disease burden, and the emigration of skilled health workers [8, 22].

Over the last year, there has been an increase in the out-migration of nurses, leading to burnout and a need for more access to qualified health workers in rural Fiji [8]. Our study found that the shortage of nurses in primary healthcare settings poses a challenge to the supervision process. This shortage is likely due to the ongoing migration of health workers from Pacific Island countries to Australia and New Zealand, which continues to affect the accessibility and quality of healthcare, particularly at the primary care level [8].

Having trained CHWs who are responsive and accountable to their communities and the health system can potentially bridge this gap, as evidenced by a recent systematic review conducted by Scott et al. (2018) [23]. Their study found that community embeddedness, supportive supervision, continuous education, logistical support and supplies were vital for positive CHW programs [18]. Another exploratory review by Vaughan et al. (2015) highlighted the cost-effectiveness and sustainability of CHW programs in low and middle-income countries (LMICs) [24]. CHWs are especially important in Fiji as they provide a focal point to assist zonal nurses in the community.

Furthermore, the Fiji CHW program can take lessons from other CHW programs in LMICs. For example, the CHW programs in Ethiopia (the CHW referred to as Health Extension Workers) and India (Accredited Social Health Activists) have standardised pre- and post-service training [18, 20]. Both countries have a formal education requirement (10^th^ grade in rural settings) and undergo a fixed-term training curriculum with practical placements. CHWs in Ethiopia work in their communities as salaried government employees after graduation [18, 20]. They have an opportunity for career progression and can earn a Level IV qualification and have an in-service integrated refresher training program to supplement their prior training [20].

The key recommendations from our study include restructuring and standardising the pre-service training and including a competency framework and refresher training for all regions. The training would include topics such as NCD prevention and control in the community. Furthermore, there is a need to provide identity cards, certification on successful completion and avenues for upskilling and career growth. Another strong aspect was using social media platforms, such as Viber and Messenger, which can assist with regular follow-up and case management, strengthening care for the rural and remote maritime population. This is further evidenced by Braun et al.’s (2013) systematic review and Li et al.’s (2023) study showing that mobile technology and social media can help improvement of CHW services through virtual communications [25, 26].

### Limitations and strengths

This study employed qualitative methodologies to delve into the implementation nuances of Fiji's CHW training program. While these methods yielded rich, context-specific insights, incorporating quantitative data could have provided an additional layer of validation, particularly regarding training records. The use of the CHW-AIM toolkit facilitated a thorough understanding of both policy and practice, enhancing the study's rigour. However, it's important to acknowledge that the results primarily encapsulate participant perceptions from six-sub-divisions in Fiji. Consequently, while the recommendations are informed and relevant, they should be contextualised within the specificities of Fiji's healthcare landscape.

## Conclusion

This study underscores significant disparities between the Fiji's CHW training policy and its practical execution, particularly concerning regional variations. Despite comprehensive nature of the policy, our findings highlight a policy-implementation gap, wherein the CHW training varies from partially functional and non-functional. This variation is primarily attributed to inconsistency in availability, delivery, frequency, and quality of the training given to the CHWs, underscoring the need for revising and standardising the training modules and competency frameworks. Such an overhaul is essential to address the existing CHWs’ capacity gaps and ensure CHWs are effectively equipped to meet diverse community health needs, improve Fiji's primary healthcare system, and achieving universal health coverage.

These findings underscore the need for a comprehensive approach to CHW training and supervision in low-resource settings, including standardized curricula, ongoing mentorship, and adequate staffing levels to ensure quality care delivery and program sustainability. The challenges identified in Fiji are likely to be mirrored in other Pacific Island countries and similar contexts worldwide, highlighting the need for global health policy to prioritize investments in community health worker programs.

### Supplementary Information

Supplementary Material 1.

Supplementary Material 2.

## Figures and Tables

**Figure:**
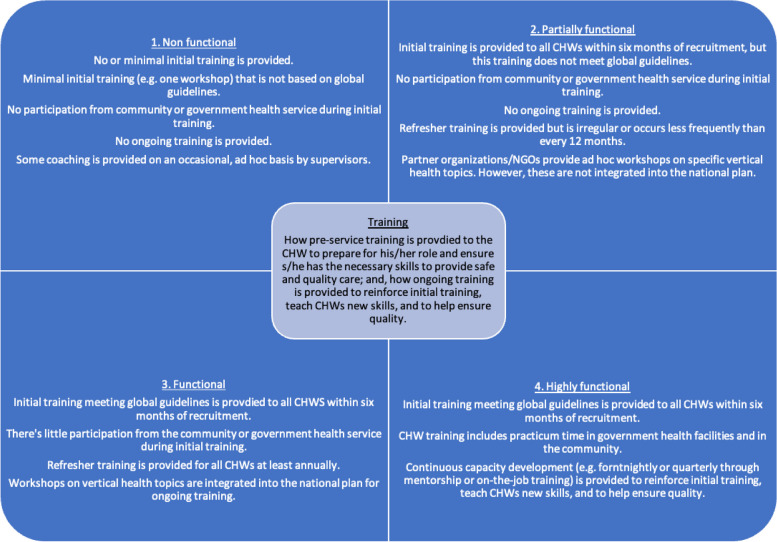
Figure adopted from the Community Health Worker Assessment and Improvement Matrix (CHW AIM) [16].

## Data Availability

All relevant data contributing to the findings are within the paper, Appendices,and Supplementary file 1.

## Appendix 1

**Table 4:** Training modules/content reported by participants and their training providers

Training Modules/Content	Eastern Division	Western Division	Central Division	Northern Division
Core Competency^a^	MOHMS	MOHMS	MOHMS	MOHMS
Safe Motherhood^a^	MOHMS	MOHMS	MOHMS	MOHMS
Child Health^a^		MOHMS	MOHMS	
Emergency^a^			MOHMS	
Palliative Care (Dressings)^a^		Cancer Society	Cancer Society	
Wellness Promotion^a^	MOHMS	MOHMS	MOHMS	MOHMS
First Aid / CPR	Red Cross	Red Cross	Red Cross	Red Cross
Vital Measurement		Red Cross	MOHMS/Cancer Society	
NCD			MOHMS	Red Cross
Tuberculosis^a^		Red Cross	MOHMS	
Ischemic Heart Disease			MOHMS	
Rheumatic Heart Disease^a^			MOHMS	
COVID-19				Red Cross

^a^*MOHMS* Ministry of Health and Medical Services training modules

## Appendix 2

**Table 5:** FGD participants’ (CHWs) characteristics (*n*=41)

Participant	Sex	Years Worked as a CHW	Age	Division	Highest Level of Education	Last Attended Training Session
P1	F	<2 years	39	Eastern	Secondary	Did not attend any training
P2	F	>6 years	49	Eastern	Secondary	2013
P3	F	2-6 years	52	Eastern	Secondary	Did not attend any training
P4	F	>6 years	55	Eastern	Secondary	2013
P5	F	2-6 years	65	Eastern	Secondary	Did not attend any training
P6	F	<2 years	43	Eastern	College	Did not attend any training
P7	F	>6 years	50	Eastern	Primary	2013
P8	F	2-6 years	55	Eastern	Secondary	Did not attend any training
P9	F	>6 years	45	Western	College	2022
P10	F	2-6 years	51	Western	College	2022
P11	M	<2 years	48	Western	Secondary	2022
P12	F	>6 years	53	Western	Secondary	2022
P13	F	>6 years	49	Western	College	2022
P14	M	<2 years	36	Western	Secondary	2022
P15	F	>6 years	47	Western	Secondary	2022
P16	F	2-6 years	30	Western	Secondary	2022
P17	F	>6 years	57	Central	Secondary	2023
P18	F	>6 years	42	Central	Secondary	2023
P19	F	>6 years	51	Central	Secondary	2023
P20	F	<2 years	37	Central	Secondary	2023
P21	F	>6 years	41	Central	Postgraduate	2023
P22	F	<2 years	37	Northern	College	Did not attend any training
P23	F	>6 years	41	Northern	Secondary	2022
P24	F	2-6 years	49	Northern	Secondary	2022
P25	F	>6 years	37	Northern	College	2022
P26	F	>6 years	53	Northern	Primary	2022
P27	F	2-6 years	39	Northern	College	2022
P28	F	>6 years	45	Northern	Secondary	2022
P29	F	<2 years	40	Northern	Secondary	2022
P30	F	>6 years	60	Northern	Secondary	2022
P31	F	>6 years	51	Central	Secondary	2023
P32	F	>6 years	44	Central	Secondary	2023
P33	F	>6 years	46	Western	Secondary	2022
P34	M	2-6 years	51	Western	Secondary	N/A
P35	F	>6 years	49	Western	College	2022
P36	F	<2 years	53	Western	Secondary	2022
P37	F	2-6 years	53	Western	College	N/A
P38	F	<2 years	38	Western	Secondary	N/A
P39	F	>6 years	50	Western	College	2022
P40	F	2-6 years	43	Western	Secondary	2022
P41	F	>6 years	60	Western	Secondary	2022

## Appendix 4

**Table 6:** Themes and quotes from the study

Themes	Quotes
**Theme 1: Variability in the Initial Training of CHWs**	*‘So, I was chosen to be, to attend the training. So, that training was for six weeks at the My Mission Hospital.’*
	*‘I [ supervisor] run the training in health centre where these people [CHWs] will all come there. For first training, we conduct once a year.’*
**Theme 2: Variability in Training Content, Duration, and Providers for CHWs**	*‘I guess there are too many training modules, like nine books. I'm sure it'll be a bit too much for them [CHWs]. It's a bit lengthy when you look at those books, the training materials because there's a participant booklet and there is a trainer’s booklet. Yeah. And it's a repetition.’*
	*‘… we teach them [CHWs] on seven areas or modules. The first one competency, the work that they can do.’*
	*‘I took the training given by the Ministry of Health.’*
**Theme 3: Supervision as a Means for Capacity Building and Evaluation**	*‘We [supervisors] evaluate the CHWs first through their performance. Through the report they submit. Then we go out to do outreach.’*
	*‘…we usually have chat groups, we usually put all our questions/requests in that group for the nurses.’*
	*‘Every month, I [supervisor] receive the report. If I see that they are not doing what is expected of them, I used to wait for them the next month that they bring their report, and we’ll talk about it.’*
**Theme 4: Recommendations from Participants**	*‘The CHW program need be strengthened so that we work on communicable and non-communicable diseases and early referral.’*
	*‘My wish list, like I did all this training, I’m looking forward to becoming just like a part-time nurse or something. But I need more training. I wanted to become a nurse, it was my dream, but I didn’t, my parents cannot afford me to do further studies so I couldn’t.’*
	*‘…we need more trainings because we are not educated like the health, the nurses, eh? So, we need to be reminded every time, so that in spite of all that work, we don’t forget sometimes, things that we focus on.’*
	*‘…We need a certificate for all of us to be recognised.’*
