# Supplementary material for: ‘First of all, I need training’: a qualitative study evaluating the Fiji community health worker training program
Source: BMC Prim Care. 2024 Jun 26;25:228. doi: 10.1186/s12875-024-02480-8 (PMC11201366; doi:10.1186/s12875-024-02480-8)
Supplement: Supplementary file 1 — Supplementary Material 1. [file 12875_2024_2480_MOESM1_ESM.pdf]

Fiji Community Health Workers FGDs guide

The aim of the meeting

This Focus Group discussion (FGD) will be conducted to understand the current role of the CHWs in delivering primary health care to the community. We would like to know more about the activities that you perform and the challenges that you face in delivering care.

1. Recruitment and role of CHWs

- How were you recruited to be CHW? (Community, government, NGO/CBO, Other (please explain)
- How were you assigned to the community(s) in which you currently work?
- Tell us about your current role as a CHWs? What are your responsibilities?
- Can you tell us about your experience in providing services for the community?
- What are the main challenges that you face while delivering health care, support and awareness?
- Did you talk to patients about their lifestyle risk factors? If so how did they respond to your advice?

2. Training

- Did you receive any initial training to prepare you for your role? Were you evaluated before or after this training?
- Have you been trained for chronic diseases management?
- A new program for hypertension and diabetes control is being launched, how do you think you can contribute towards this role.
- Do you usually receive any additional training (refresher/ongoing training) to help you fulfil your role? Do you get evaluated after receiving training?
- Who do you contact if something is unclear to you while doing your job or if you encounter any challenges in the community?

3. Equipment and Supplies

- Do you have the supplies and equipment you need to provide the services you are expected to deliver?
- Do you dispense any medicines? If you have experienced shortages or stock outs of supplies, how do you get more supplies?
- Probe: How often do you get them? / Do you use any form(s)? / How do you get the form(s) and to whom do you submit them?

4. **Supervision**

- Could you tell us about the formal supervisory mechanism? How often does your supervisor visit you?
- What does your supervisor do when he/she visits you? Probe: Observation of service delivery, Coaching and skills development, Trouble shooting, problem solving, Record Review, Supply check
- Do they usually accompany you in field visits or you meet them at the health centre?
- Does your supervisor provide you any feedback regarding your performance?

End of session questions

- Is there anything else that you would like to share with me?

Interview guide (Policy makers / Supervisors)

Aim of the interview

This interview is part of a research study "Assessing the role of community health workers in Primary Health Care in Fiji".

EXPLORATORY QEUSTIONS:

1. Role of CHWs overall and for CVD service delivery

Can you please tell us about the role of the CHWs? Is there any available guidelines for their role?

How do the CHWs get introduced to their job role?

How do the CHWs get feedback regarding their role? Any guidance?

Is their role and task(s) clearly understood within the community?

How do you think the PEN program can be introduced to the CHW program?

2. Training

Can you please tell us more about the available training provided to CHWs?

Any special guidelines for the NCD management training?

How are they trained and evaluated? When PEN is integrated into the CHW program, how will the CHWs be trained to deliver quality health care?

Can you please tell us about the continuous training of the CHWs, are all the CHWs included or do you follow special guidelines to select the CHWs for training?

Do the current modules for training include NCD training for CHWs?

How do CHWs look after people with NCDs living in their community – how do they support them?

Do you feel that CHWs have the capacity, skills and equipment to look after patients with NCDs in their community?

3. Equipment and Supplies

Can you please tell us more about the equipment and supplies provided for the CHWs?

As MHMS is planning to roll out PEN, how will the equipment and infrastructure be maintained to deliver care for CVD?

4. <u>Supervision</u>

Can you please tell us about the supervision of CHWs? What is the process for supportive supervision?

Can you tell us about supervision checklists or any other supervision tools that exist to help guide supervisors?

5. <u>Individual Performance Evaluation</u>

Can you please tell us about the process for conducting individual performance evaluations for CHWs? Who is usually involved in the evaluation process?

6. <u>Incentives</u>

Can you please tell us about the available guidelines for the financial or non-financial incentives provided to CHWs? Are these guidelines based on performance evaluation?

Are there any extra incentives for the NCD management role?

8. <u>Opportunity for Advancement/ CAREER PROGRESSION</u>

Can you please tell us about the available advancement opportunities for CHWs?

How do the CHWs usually get informed about these opportunities?

<u>End of session questions</u>

- Is there anything else that you would like to share with me?
